# Characterization of the fatty acid metabolism-related genes in lung adenocarcinoma to guide clinical therapy

**DOI:** 10.1186/s12890-022-02286-3

**Published:** 2022-12-23

**Authors:** Guichuan Huang, Jing Zhang, Ling Gong, Xiaowen Wang, Bangyan Zhang, Daishun Liu

**Affiliations:** 1 State Key Laboratory of Respiratory Disease and National Clinical Research Center for Respiratory Disease, The First Affiliated Hospital of Guangzhou Medical University, Guangzhou, 510000 China; 2 Department of Pulmonary and Critical Care Medicine, Affiliated Hospital of Zunyi Medical University, Zunyi, 563000 China; 3 Department of Pulmonary and Critical Care Medicine, The First People’s Hospital of Zunyi (The Third Affiliated Hospital of Zunyi Medical University), No 98 Fenghuang Road, Huichuan District, Zunyi, 563000 China; 4 Department of Respiratory and Critical Care Medicine, Guizhou Provincial People’s Hospital, Guiyang, 550000 China

**Keywords:** Lung adenocarcinoma, Fatty acid metabolism, Prognosis, Risk score model

## Abstract

**Background:**

Lung adenocarcinoma (LUAD) is a common cancer with a bad prognosis. Numerous investigations have indicated that the metabolism of fatty acids plays an important role in the occurrence, progression, and treatment of cancer. Consequently, the objective of the current investigation was to elucidate the role and prognostic significance of genes associated with fatty acid metabolism in patients diagnosed with LUAD.

**Materials and methods:**

The data files were acquired from The Cancer Genome Atlas database and GSE31210 dataset. Univariate Cox and least absolute shrinkage and selection operator regression analyses were conducted to establish a prognostic risk scoring model depending on fatty acid metabolism-associated genes to predict the prognosis of patients with LUAD. pRRophetic algorithm was utilized to evaluate the potential therapeutic agents. Gene set variation analysis combined with cell-type identification based on the estimation of relative subsets of RNA transcript and single-sample gene set enrichment analysis was used to determine the association between immune cell infiltration and risk score. Tumor immune dysfunction and exclusion algorithm was employed to predict immunotherapeutic sensitivity.

**Results:**

To forecast the prognosis of patients with LUAD, a risk scoring model based on five genes associated with fatty acid metabolism was developed, including *LDHA*, *ALDOA*, *CYP4B1*, *DPEP2*, and *HPGDS*. Using the risk score algorithm, patients were divided into higher- and lower-risk categories. Patients classified as minimal risk showed superior prognosis than those with elevated risk. In addition, individuals in the higher-risk group had a proclivity toward chemoresistance and amenable to immunotherapy.

**Conclusion:**

The prognostic risk scoring model aids in estimating the prognosis of LUAD patients. It may also provide new insights into LUAD carcinogenesis and therapeutic strategies.

**Supplementary Information:**

The online version contains supplementary material available at 10.1186/s12890-022-02286-3.

## Introduction

Lung cancer is the most frequent type of cancer and the greatest cause of mortality from cancer on a global scale [1]. In men and women, adenocarcinoma of the lung (LUAD) is the most prevalent lung cancer, contributing to 40% of all cases of lung cancer [2, 3]. Although clinical advances in early identification and focused therapy have been established, the 5-year survival rate for LUAD remains poor [4]. Therefore, the elucidation of the molecular mechanism and identification of reliable prognostic biomarkers are very important for the treatment and prognosis of patients with LUAD.

Aberrant metabolic reprogramming of energy is a significant element in the onset and progress of cancer. For instance, an upregulation of glycolysis, glycogen metabolism, and gluconeogenesis was observed in cancer cells, which is known as “Warburg effect” [5]. In addition, an abundant supply of amino acids is indispensable for cancer cell growth [6]. In recent years, abnormal fatty acid metabolism in cancer cells has received increased attention. For instance, Liang et al. reported that *ACOT11* regulates tumor proliferation and invasion by binding with *CSE1L* in LUAD [7]. *ACSL4* is a long-chain acyl-coenzyme synthetase and participates in fatty acid biosynthesis and catabolism. Zhang et al. [8] demonstrated that *ASCL4* inhibits tumor cell survival, invasion, and migration and promotes ferroptosis in LUAD. However, there has been no extensive investigation of fatty acid metabolism-related genes in LUAD.

The genes associated with fatty acid metabolism contributing to the prognosis of patients with LUAD were analyzed. Using data from The Cancer Genome Atlas (TCGA), a prognostic risk scoring model was constructed to further evaluate the GSE31210 dataset. Patients with LUAD can be classified into higher- and lower-risk groups based on the median risk score. Lastly, the characteristics, treatment, and immune cell infiltration in lower- and higher-risk groups were also studied. This study facilitates the investigation of the metabolic process and targeted therapy for LUAD.

## Materials and methods

### Data collection

The LUAD RNA-seq data profile was retrieved from the TCGA database (535 LUAD samples and 59 normal lung samples) (https://portal.gdc.cancer.gov/). Additionally, clinical data of individuals with LUAD were extracted from the TCGA database (522 patients). There were 513 matched LUAD patients between RNA-seq data file and clinical findings (Additional file 1: Table S1). Additionally, the Gene Expression Omnibus (GEO) database was queried for GSE31210, an RNA-seq data profile of LUAD (http://www.ncbi.nlm.nih.gov/geo/) (20 normal lung samples and 226 LUAD samples). The clinical information of 226 patients with LUAD in GSE31210 is displayed in Additional file 1: Table S2.

In a previous study [9], we reported a total of 309 genes involved in the metabolism of fatty acids (Additional file 1: Table S3).

### Identification of differentially expressed genes associated with fatty acid metabolism

The differentially expressed genes (DEGs) associated with fatty acid metabolism were compared between normal and malignant tissues utilizing the R package “limma” with **|**log_2_[fold change (FC)] **|**> 1 and false discovery rate (FDR) < 0.05. The R package “pheatmap” was utilized to display the findings.

### Construction and validation of the prognostic risk score model

As a training set, the LUAD data in the TCGA group were employed. The LUAD data in GSE31210 dataset was used as the test set. First, we explored the prognosis-related genes from fatty acid metabolism-associated DEGs using a univariate Cox regression model. If *p* < 0.05, the genes were retained. In addition, we analyzed the gene mutations in the LUAD samples from the TCGA cohort using the R package of “map tools”. To refine the selection of critical genes associated with fatty acid metabolism for prognostic risk assessment, the least absolute shrinkage and selection operator (LASSO) regression analysis was utilized. The following risk score formula was used: Risk score = expression of gene 1 × β1 + expression of gene 2 × β2$+ \cdots +$ expression of gene n × βn. β reflects the regression coefficient of the associated gene based on the LASSO regression analysis. All LUAD samples were categorized into lower- and higher-risk cohorts based on the median value of the risk score. To analyze the difference in survival between lower- and higher-risk groups, a log-rank test and Kaplan–Meier analysis were conducted.

To assess the predictive accuracy of prognostic risk scoring model, a receiver operating characteristic (ROC) curve analysis was performed. Lastly, the prognostic model for risk score was validated using the GSE31210 dataset.

### Principal component analysis before and after risk score prognosis

First, depending on the genes involved in fatty acid metabolism, we utilized the R package of “limma” to perform the principal component analysis (PCA) of sample distribution between the lower- and higher-risk cohorts. Then, depending on the genes identified in the prognostic risk score model, PCA was performed again. Finally, we displayed the findings of PCA using the R package of “ggplot2”.

### Relationship between clinical parameters and risk scores

The relationship between risk scores and clinical parameters including gender, age, and TNM stage was determined. Based on the difference in risk scores, the LUAD samples were separated into distinct groups.

### Gene set variation analysis

Gene set variance analysis (GSVA) is an unsupervised approach used to determine the variation in pathway activity across a sample population [10]. Therefore, to investigate the biological processes between higher-risk and lower-risk groups, GSVA was conducted using the “GSVA” R package. The gene set of “c2.cp.kegg.v7.4.symbols” downloaded from the molecular signature database (MSigDB) was used as the reference gene set. FDR < 0.05 was regarded as a statistically significant enrichment pathway.

### Characteristics based on risk stratification

To predict the drug sensitivity of chemotherapy and targeted therapy in the two risk groups of LUAD, the half-maximal (IC50) inhibitory concentration of drugs was determined utilizing the “pRRophetic” R package [11]. Additionally, we identified cell types by the calculating relative subsets of RNA transcripts (CIBERSORT) to investigate immune cell infiltration in every LUAD sample obtained from the higher- and lower-risk groups. CIBERSORT is a computational approach used to measure immune cell fractions based on gene expression patterns in bulk tissues derived from RNA-sequence analysis [12]. The study demonstrated that CIBERSORT was effective in reliably estimating the immune cell landscapes of a variety of malignancies [13]. The gene sets were obtained from a prior study in order to investigate immune-related activities in the tumor microenvironment (TME) [14–16]. The immune-related activity was scored among the higher- and lower-risk groups via single-sample gene set enrichment analysis (ssGSEA), such as co-inhibition and co-stimulation of T cells. Finally, the tumor dysfunctional immune system and exclusion (TIDE) score (http://tide.dfci.harvard.edu) was utilized to evaluate the potential response to immunotherapy in patients with LUAD in both risk categories [17]. In general, a lower TIDE score indicated improved immunotherapy response.

### Screening of DEGs in the higher- and lower-risk groups for GO and KEGG analyses

The DEGs in higher- and lower-risk groups were obtained utilizing the “limma” R package. Genes with the |log_2_FC|> 1 and FDR < 0.05 were considered as DEGs. The gene ontology (GO) and Kyoto Encyclopedia of Genes and Genomes (KEGG) enrichment analyses of DEGs were performed utilizing the R package of “clusterProfiler”.

### Statistical analysis

The statistical difference in distribution (gene expression and scores) between the two groups was compared using the Wilcoxon test, and between three or more groups via Kruskal–Wallis test. Univariate Cox regression analysis and LASSO regression analysis were performed to determine the prognosis-related genes. Kaplan–Meier analysis with log-rank test was conducted to determine the overall survival (OS) and progression-free survival (PFS) of the two risk categories. *P* < 0.05 was considered statistically significant.

## Results

### Identification of fatty acid metabolism-associated DEGs in patients with LUAD

**Fig. 1:**
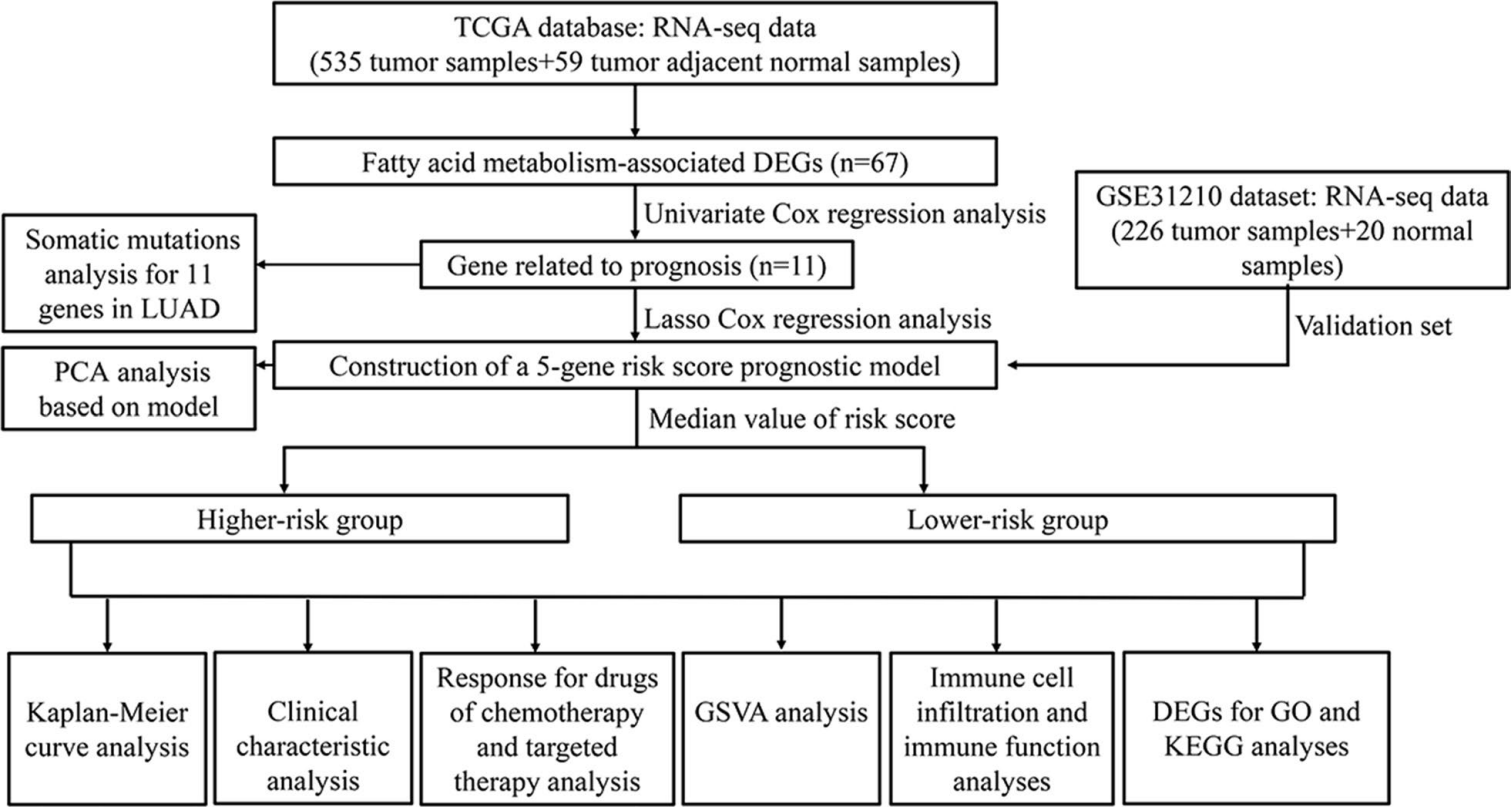
The flowchart for this investigation

**Fig. 2:**
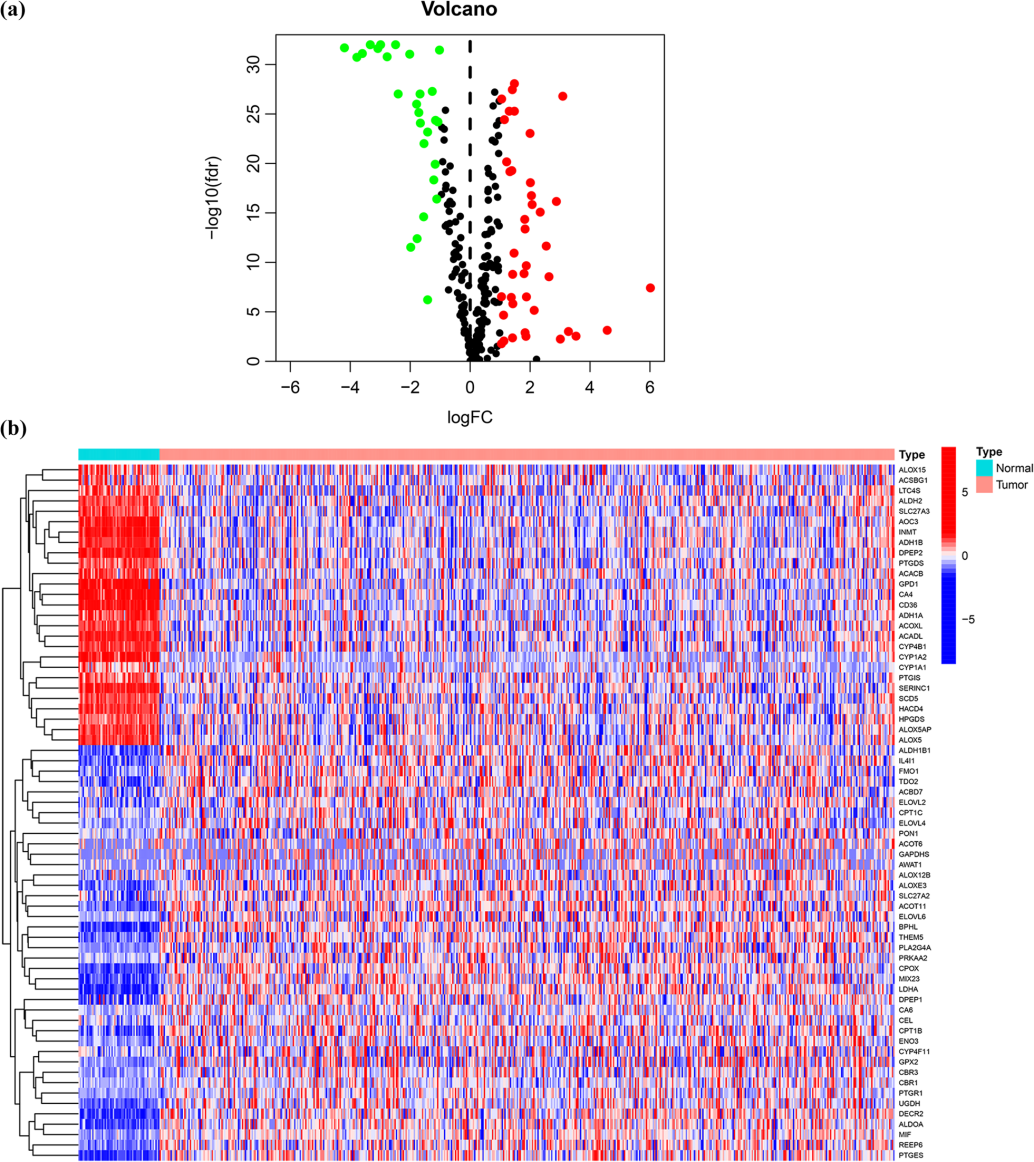
Identification of fatty acid metabolism-associated DEGs. **a** Volcano map of DEGs in LUAD and normal tissue; **b** The heatmap of DEGs in normal tissue and LUAD tissue

Figure 1 presents the comprehensive flowchart of this investigation. We analyzed the expression of fatty acid metabolism-associated genes between the LUAD tissue sample and normal lung tissue in the TCGA cohort. Of the 309 genes associated with fatty acid metabolism, 67 were DEGs, which included 27 downregulated genes and 40 upregulated genes in LUAD tissue samples (Fig. 2A, B).

### Construction and assessment of a prognostic risk scoring model based on selected fatty acid-metabolism-associated genes

**Fig. 3:**
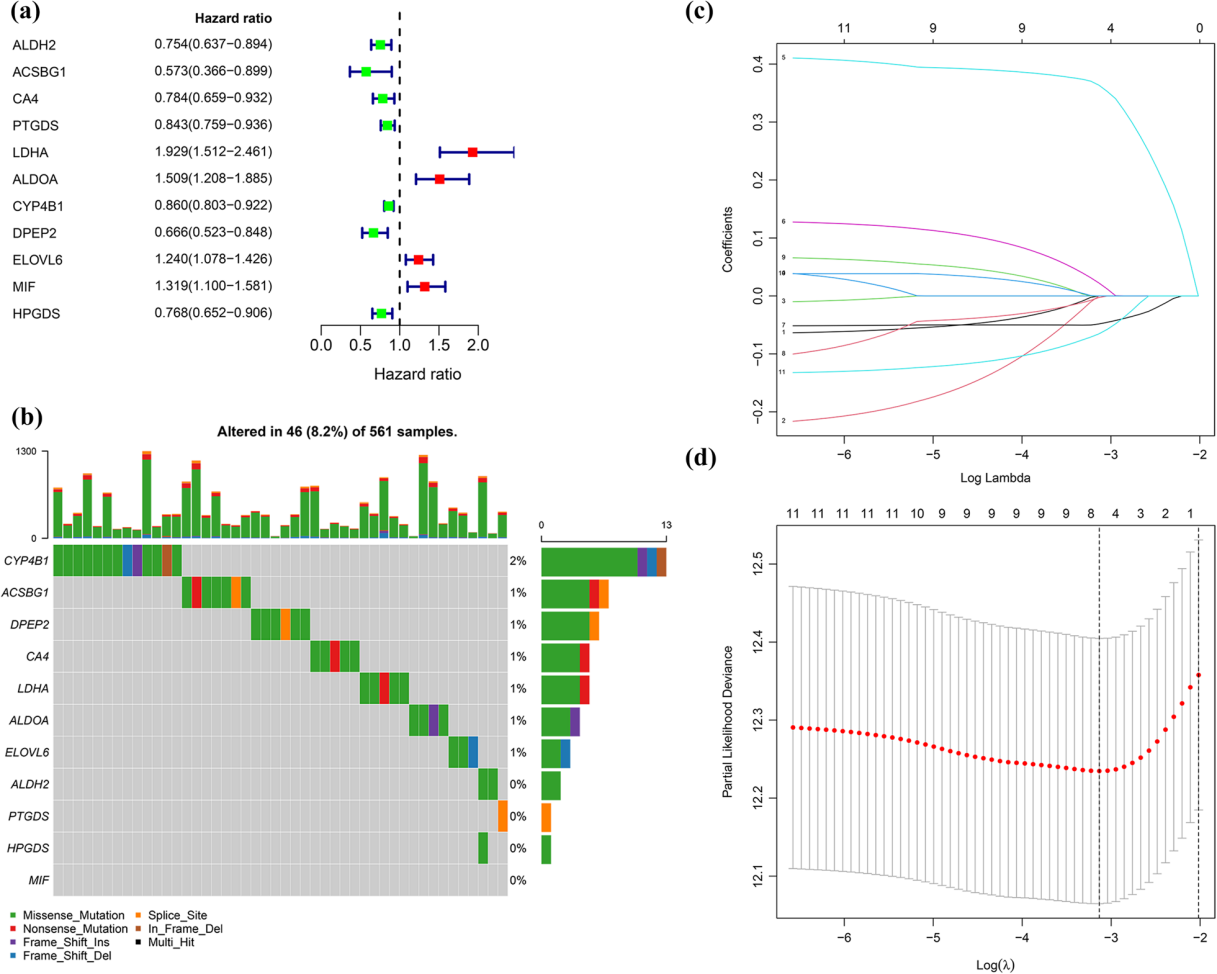
Construction of a risk score prognostic model. **a** The forest plot of eleven genes involved in fatty acid metabolism; **b** Eleven fatty acid metabolism-associated genes mutation rate in 561 LUAD samples from the TCGA database; **c** LASSO coefficients of the eleven fatty acid metabolism-associated genes; **d** Cross-validation for the purpose of identifying critical genes for the predictive method based on the risk score

Samples obtained from the TCGA cohort were used as the training set. Of the 513 patients with LUAD in the TCGA group, only 504 were included in the survival analysis because of missing survival times involving 9 patients. First, a univariate Cox regression analysis was of 67 DEGs involved in fatty acid metabolism was performed. We obtained 11 genes related to prognosis (*p* < 0.05). Among the 11 genes, 7 genes (*ACSBG1*, *DPEP2*, *ALDH2*, *HPGDS*, *CA4*, *PTGDS*, and *CYP4B1*) were negatively related to prognosis because of 0 < hazard ratio (HR) < 1, whereas 4 genes (*ELOVL6*, *MIF*, *ALDOA*, and *LDHA*)were positively related to prognosis based on HR > 1 (Fig. 3A). We then analyzed the somatic mutations of 11 fatty acid metabolism-associated DEGs related to prognosis in 561 LUAD samples from the TCGA database. The findings revealed that 46 LUAD samples (8.2%) carried mutations involving fatty acid metabolism-associated genes (Fig. 3B). *CYP4B1* showed the highest mutation frequency in LUAD samples, whereas no *MIF* mutations were found in LUAD samples (Fig. 3B).

Initially, LASSO regression analysis was conducted to determine the key genes among the above 11 fatty acid metabolism-associated DEGs related to prognosis (Fig. 3C, D). Finally, five genes (*LDHA*, *ALDOA*, *CYP4B1*, *DPEP2*, and *HPGDS*) were used to construct the prognostic risk scoring model according the following formula: Risk score = *LDHA* expression × (0.363478498) + *ALDOA* expression × (0.022162053) + *CYP4B1* expression × (− 0.04831086) + *DPEP2* expression × (− 0.002618813) + *HPGDS* expression × (− 0.065320579). The details of the five genes are displayed in Additional file 1: Table S4. The expression of the five genes in normal lung and LUAD tissues of the TCGA group and GSE31210 dataset is illustrated in Additional file 1: Figure S1.

**Fig. 4:**
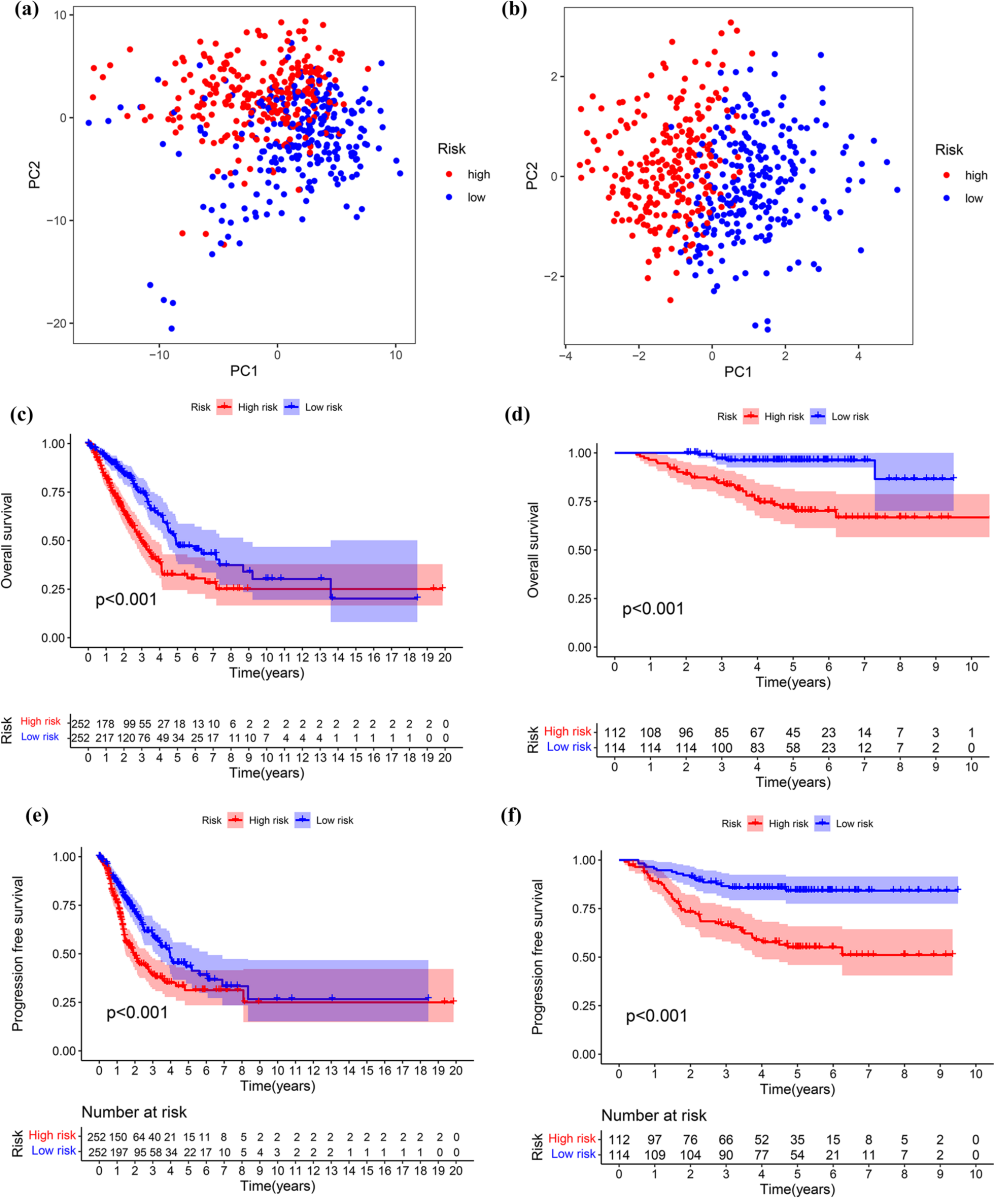
The prognostic value of risk score model. **a** Principal component analysis depended basically on all genes involved in fatty acid metabolism in LUAD; **b** Principal component analysis based upon 5 fatty acid metabolism-associated genes from risk score prognostic model; **c** Kaplan–Meier curves for the overall survival of patients between the higher-risk and lower-risk groups in the TCGA cohort; **d** Kaplan–Meier curves for the overall survival of patients between the higher-risk and lower-risk groups in the GSE31210 dataset; **e** Kaplan–Meier curves for the progression free survival of patients between the higher-risk and lower-risk groups in the TCGA cohort; **f** Kaplan–Meier curves for the progression free survival of patients between the higher-risk and lower-risk groups in the GSE31210 dataset

As a cut-off number, based on the median value of risk scores in the TCGA, patients with LUAD were stratified into lower risk (n = 252) and higher risk (n = 252). First, PCA was conducted to identify patients at higher and lower risk. As demonstrated in Fig. 4A, B, the prognostic risk scoring model can be used to distinguish LUAD in different risk groups. The Kaplan–Meier curve analysis of the TCGA cohort revealed that individuals at lower risk had longer OS and PFS than those at higher risk (Fig. 4C, E). To establish the accuracy of the prognostic risk scores, they were applied to patients in the GSE31230 dataset based on the TCGA cut-off value. As illustrated in Fig. 4F, D***,*** the higher-risk cohort (n = 112) had a worse OS and PFS than the lower-risk cohort (n = 114). The prognostic risk scoring model accurately predicted the outcome in patients with LUAD.

### Risk score is an independent prognostic indicator

**Fig. 5:**
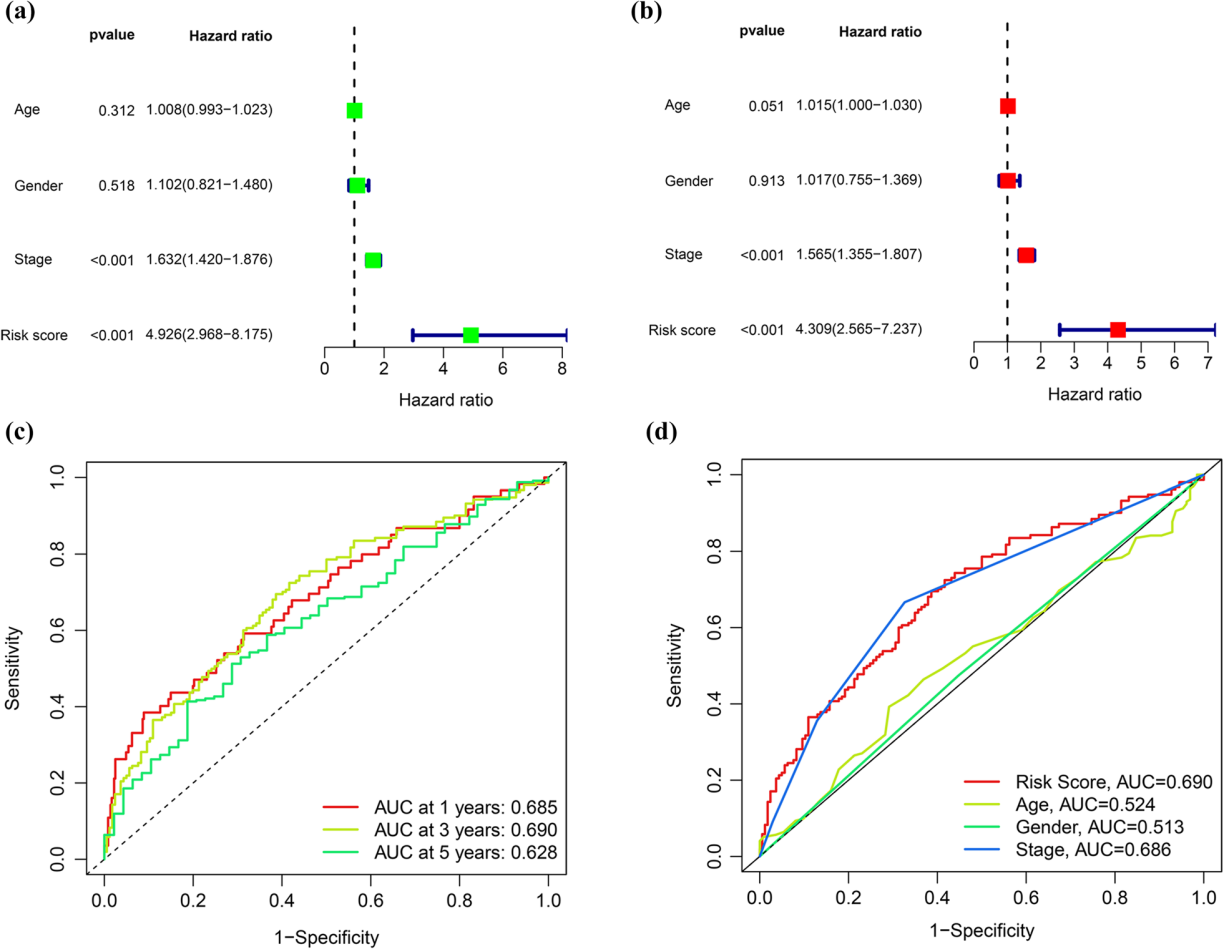
Identification independent prognostic factors in LUAD. **a**, **b** Analysis of Cox regression, both univariate and multivariate, for clinical parameters and risk scores model; **c** ROC curves of risk score model at one, three, and 5 years of overall survival; **d** ROC curves for clinical parameters and a model of the risk score

Cox regression analysis (univariate and multivariate) was used to determine whether the risk score was a significant and independent predictor of LUAD. Numerous clinicopathological factors were analyzed, including gender, stage and age. The univariate analysis revealed that stage and risk score were associated with prognosis (Fig. 5A). Additionally, subsequent multivariate analysis demonstrated that risk score and stage were related to survival (Fig. 5B). These data indicate that the risk score may be utilized as a stand-alone prognostic factor for LUAD.

The values of area under the ROC curve (AUC) for the risk scores at one, three, and 5 years of OS were 0.685, 0.690, and 0.628, respectively (Fig. 5C). Additionally, the AUC value revealed that risk score had a better prognostic value than age, gender, and stage (Fig. 5D).

### Relationship between risk scores and clinicopathological features

**Fig. 6:**
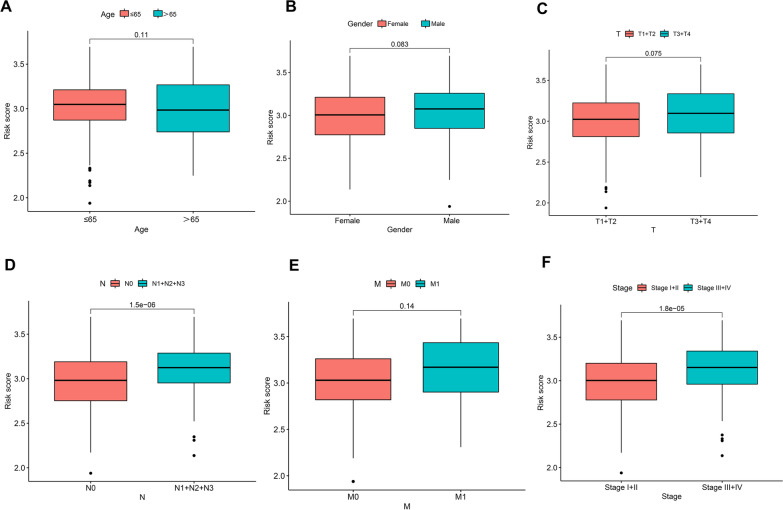
Association between risk scores and clinicopathological parameters, including age (**A**), gender (**B**), T stage (**C**), N stage (**D**), M stage (**E**), and TNM stage (**F**)

To determine the relationship between risk scores and clinical characteristics, the risk score distributions based on age, gender, and T, N, and M stages were analyzed. There was no significance in risk scores associated with gender, age, T, and M. However, greater risk scores were associated with lymphoid metastases and advanced stage of cancer (Fig. 6).

### Drug response to chemotherapy and targeted therapy

**Fig. 7:**
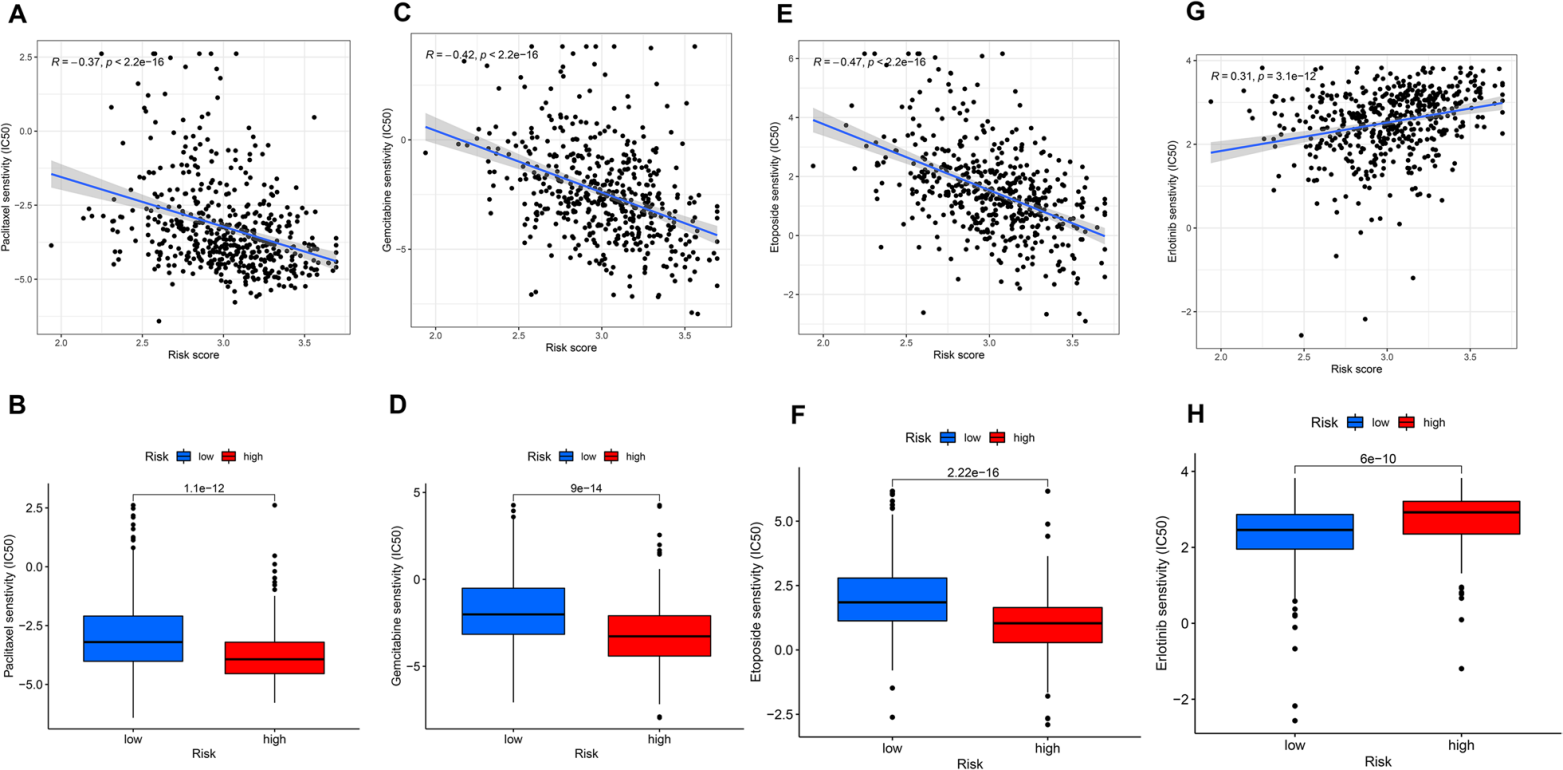
The role of risk score model in chemotherapy and targeted therapy. The correlation between risk scores and IC50 value of paclitaxel (**A**), gemcitabine (**C**), etoposide (**E**), and erlotinib (**G**); The drug response of paclitaxel (**B**), gemcitabine (**D**), etoposide (**F**), and erlotinib (**H**) between the higher-risk and lower-risk groups

The “pRRophetic” package was employed to evaluate the drug sensitivity of LUAD to investigate the variation in sensitivity between patients with lower and higher risk. We selected a single targeted drug therapy (erlotinib) and three chemotherapy drugs (gemcitabine, paclitaxel, and etoposide), which are widely utilized in clinical practice for the treatment of LUAD. The chemotherapy drugs were negatively correlated with risk scores and showed higher IC50 among patients at lesser risk (Fig. 7). However, erlotinib was positively correlated with risk scores and had a higher IC50 in the higher-risk group. The findings suggest that patients in the lower-risk cohort were highly vulnerable to chemotherapy, whereas targeted treatment was appropriate for those in the higher-risk cohort.

### GSVA

**Fig. 8:**
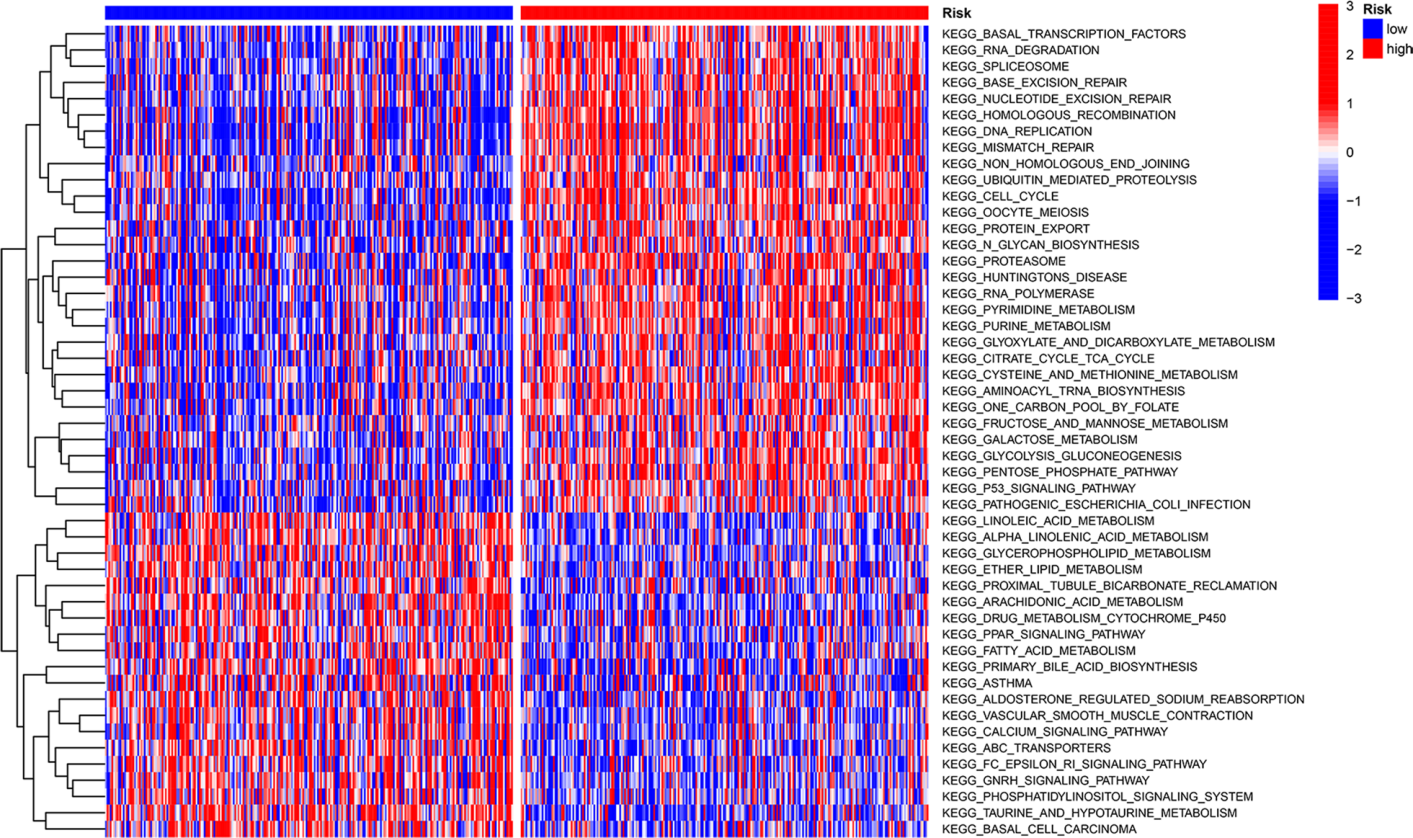
GSVA enrichment analysis between the high-risk and low-risk groups

GSVA was utilized to compare the pathway activity among the lower- and higher-risk categories in the TCGA group. The results showed that a total of 76 pathways were statistically significant. The top 50 pathways are displayed in the heatmap (Fig. 8). Interestingly, the lower-risk group had an abundance of metabolic pathways, comprising arachidonic acid, linoleic acid, fatty acid and glycerophospholipid metabolism.

### Immune cell infiltration in the risk groups

**Fig. 9:**
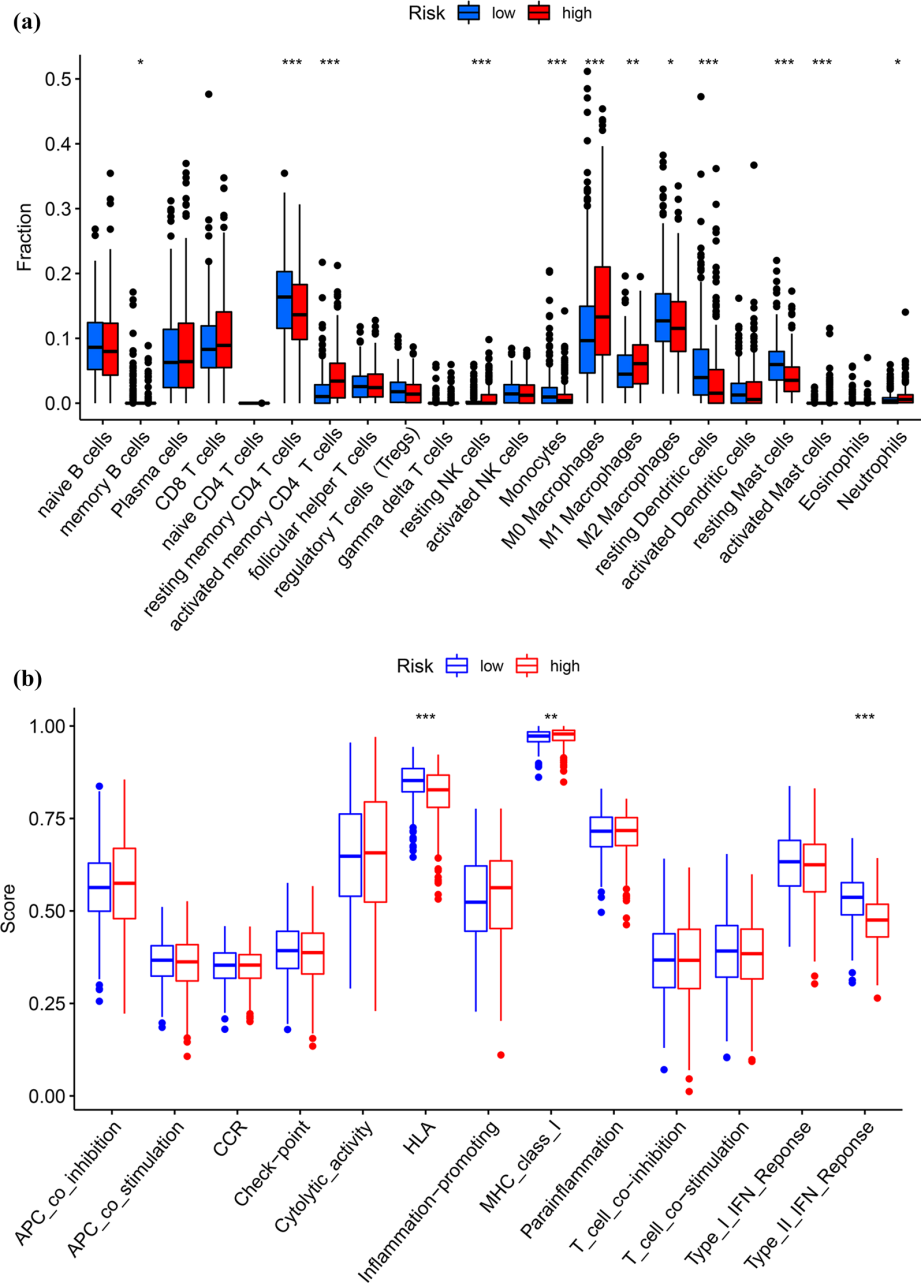

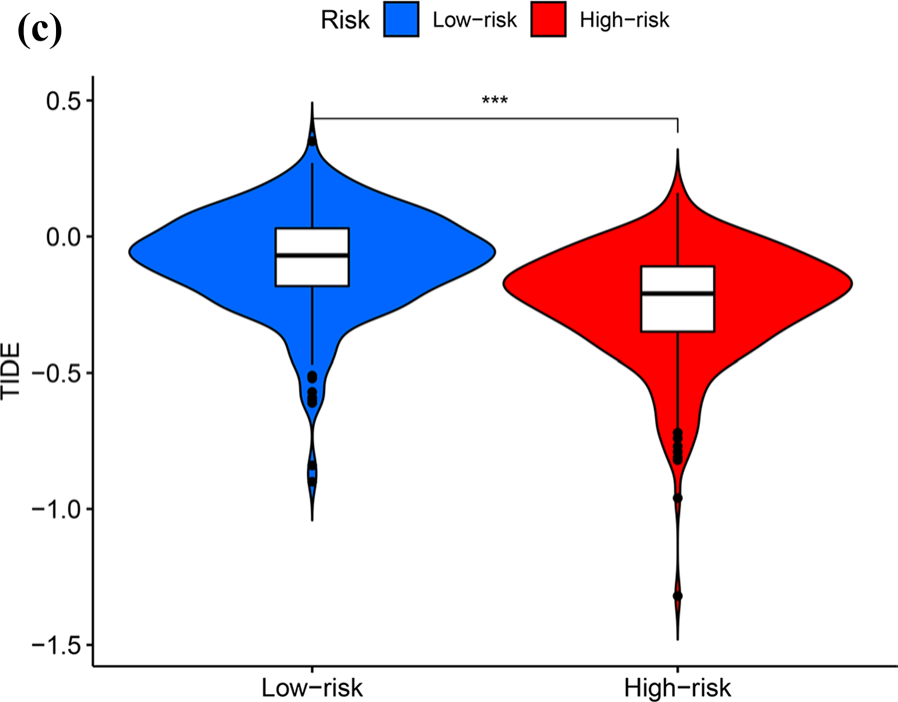
The role of the risk score model in immunotherapy. **a** Twenty-two distinct kinds of immune cells seen in the tumor microenvironment in higher- and lower-risk groups; **b** The differences of common function for immunity regulation between the higher-risk and lower-risk groups; **c** TIDE scores in the higher-risk and lower-risk groups

The infiltration of 22 immune cells in the risk cohorts was evaluated according to the CIBESORT deconvolution algorithm. The lower-risk group had less activated memory CD4 T cells, M0 macrophages, resting NK cells, active mast cells, M1 macrophages, and neutrophils than the higher-risk group (Fig. 9A). By contrast, resting dendritic cells, memory B cells, resting memory CD4 T cells, monocytes, M2 macrophages and resting mast cells in the lower-risk cohort were substantially higher than in the higher-risk cohort (Fig. 9A). Additionally, the immunological function in the higher-risk group was decreased compared with the lower-risk group, based on human leukocyte antigen (HLA) and Type II IFN response (Fig. 9B). Finally, the TIDE score in two groups was calculated to assess the effectiveness of immunotherapy (PD-1 inhibitor and CTLA-4 inhibitor) using an online TIDE database. The results suggested that patients at lower risk had elevated TIDE scores than those at higher risk, implying that higher-risk individuals may be more amenable to immunotherapy (Fig. 9C).

### GO and KEGG analyses of DEGs in the higher- and lower-risk groups

**Fig. 10:**
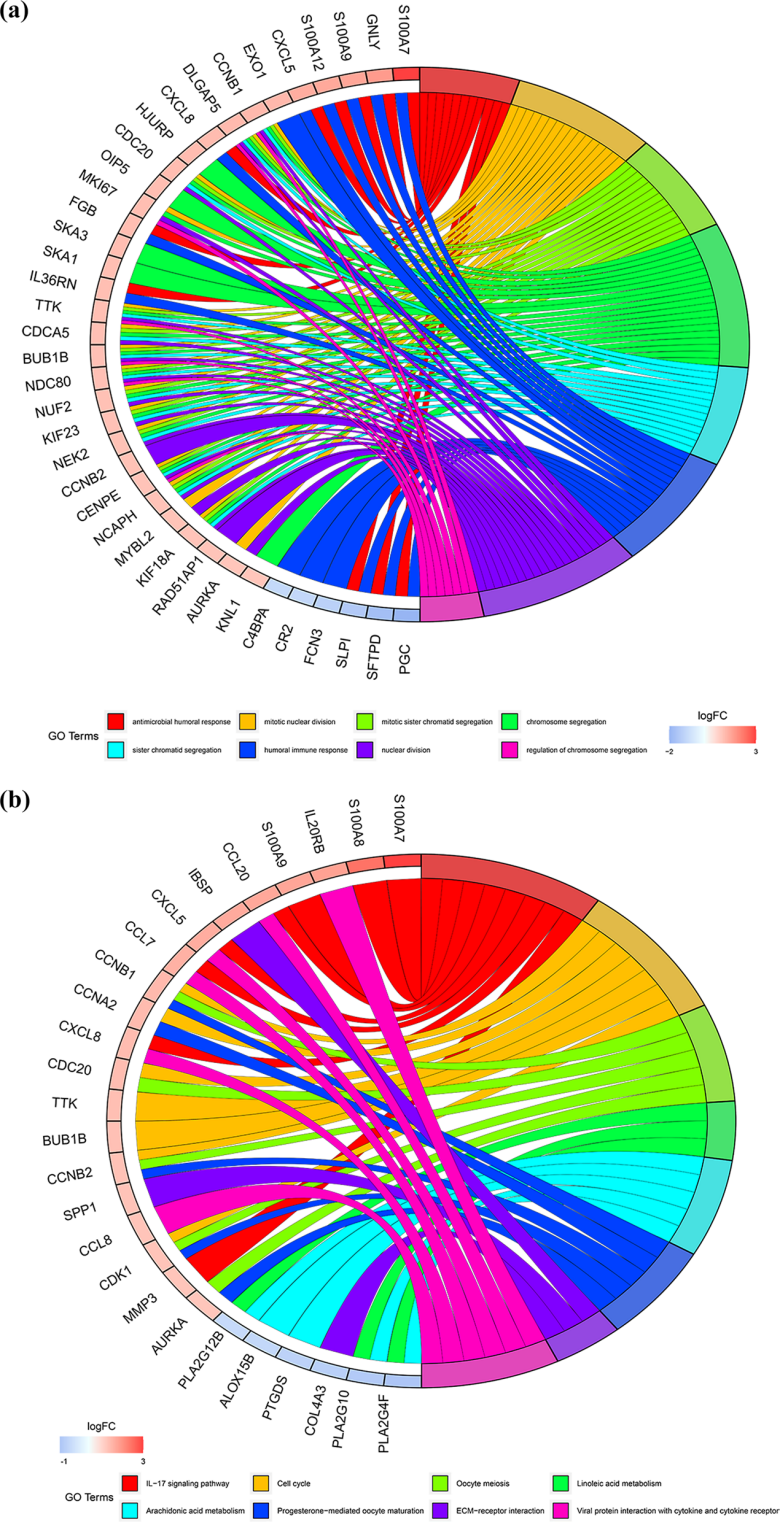
GO and KEGG analyses for DEGs between the high-risk and low-risk groups. **a** Analysis of GO enrichment for DEGs; **b** Analysis of KEGG enrichment for DEGs

To investigate the difference between the two groups, the DEGs in the higher- and lower-risk groups were determined. We obtained 504 DEGs. Among the 504 DEGs in the higher-risk group, 291 and 213 genes were downregulated and upregulated, respectively. GO and KEGG analyses of these 504 DEGs were conducted. GO analysis revealed that DEGs were significantly enriched during mitotic sister chromatid segregation, antimicrobial humoral response, nuclear division, humoral immune response, chromosome segregation, mitotic nuclear division and regulation of chromosome segregation (Fig. 10A). KEGG analysis revealed that DEGs were abundant in pathways associated with fatty acid metabolism, particularly arachidonic acid and linoleic acid metabolism (Fig. 10B).

## Discussion

Aberrant metabolic reprogramming is associated with initiation and progression of cancer [18]. Studies have indicated that metabolism-associated genes are reliable prognostic biomarkers in cancer. For instance, a nine-gene amino acid metabolism-related risk signature was utilized to predict the prognosis of patients with hepatocellular carcinoma [19]. Zhang et al. reported that a nine glycolysis-related gene signature effectively predicted metastasis and survival in patients with LUAD [20]. However, the characteristics of genes involved in fatty acid metabolism in LUAD are not fully understood.

To determine the prognosis of patients with cancer, we conducted a comprehensive analysis of genes involved in fatty acid metabolism associated with LUAD. First, depending on RNA-seq data of the TCGA group, 67 fatty acid metabolism-associated DEGs with strict filter conditions between LUAD and normal lung tissues were obtained. Second, we selected the five OS-related genes (*LDHA*, *ALDOA*, *CYP4B1*, *DPEP2*, and *HPGDS*) from DEGs utilizing univariate Cox and LASSO regression analysis to develop a predictive model of risk score. Further, the GSE31210 dataset was utilized for predictive risk assessment. After integrating with clinical dimensions, the model for predictive risk assessment was established as an adequate and effective prognostic indicator. Finally, utilizing the risk score approach, patients with LUAD were divided into higher- and lower-risk categories. The differences between higher- and lower-risk categories, including clinical parameters, chemotherapy drug susceptibility, targeted therapy, immunotherapy, and infiltration of immune cells were analyzed.

*LDHA* is a member of lactate dehydrogenase, which catalyzes pyruvate to lactate during aerobic glycolysis [21]. Evidence suggests that *LDHA* participates in fatty acid synthesis [22]. Overexpression of *LDHA* has been established in a number of malignancies, including hepatocellular carcinoma [23], breast cancer [24], and gastric cancer [25]. Additionally, investigations have shown that the expression of *LDHA* was also upregulated in LUAD; the upregulation of *LDHA* is a strong predictor of low survival in patients with LUAD [21, 26]. *ALDOA* is a crucial enzyme that is associated with fatty acid metabolism [27]. Numerous studies have established that *ALDOA* plays a role in cancer initiation and progression [28–30]. For instance, Dai et al. reported that *ALDOA* was highly expressed in colorectal cancer and high levels of *ALDOA* contributed to the aggressiveness and poor prognosis of colorectal cancer [28]. Our results revealed that *ALDOA* might be an oncogene in LUAD and was associated with survival in patients suffering from LUAD. *CYP4B1* is a member of the mammalian CYP4 enzyme family and plays an essential role in the oxidative metabolism of an extensive spectrum of endogenous compounds and xenobiotics [31]. *CYP4B1* was downregulated in LUAD and the expression of *CYP4B1* was negatively correlated with prognosis of patients with LUAD [31]. *HPGDS* is a type of glutathione transferase that catalyzes the isomerization of prostaglandin H2 to prostaglandin D2 [32]. *HPGDS* is relevant to the metabolism of fatty acids [33]. *HPGDS* promotes tumor cell apoptosis and inhibit invasion in lung cancer [34]. *DPEP2* is involved in the biosynthesis of leukotriene, and abnormal expression of *DPEP2* is responsible for dysregulated lipid metabolism [35, 36]. However, the effect of *DPEP2* in cancers, particularly lung cancer, is still unclear. In the current study, a decrease in *DPEP2* expression in LUAD indicated poor prognosis of patients suffering from LUAD.

To enhance the clinical management of patients with LUAD, we compared the differences in patients’ responses to common chemotherapeutic agents as well as a targeted agents in the higher- and lower-risk cohorts. It was found that the higher-risk group exhibited a low sensitivity to chemotherapeutic agents (gemcitabine, paclitaxel, and etoposide), suggesting chemoresistance. Fortunately, erlotinib, a targeted agent, appears to be effective in individuals at higher risk. Since patients in the higher-risk group might not be indicated for chemotherapy, we investigated whether immunotherapy was effective in such cases.

Immune cell infiltration of the tumor microenvironment occurs mainly in tumor proliferation and is an important prognostic indicator and determines patients’ response to immunotherapy in cancer, based on clinical trials using immune checkpoint inhibitors [37, 38]. Therefore, we comprehensively analyzed the immune cell infiltration in two groups. The results csuggested that activated memory CD4 T cells, resting NK cells, M0 macrophages, M1 macrophages, activated mast cells, and neutrophils were enriched in the higher-risk group. Activated mast cells are correlated with tumor angiogenesis and poor prognosis [39]. Elevated neutrophils increase tumor burden, which contributes to tumor progression and metastasis [40]. High levels of neutrophils might suppress the antitumor effects of T cells and NK cells [41]. Further, the immunological function was suppressed in the higher-risk group, including HLA and Type II IFN response. Therefore, individuals at higher risk of LUAD are highly amenable to immunotherapy, consistent with the prediction outcome of TIDE.

Of course, the current investigation has certain limitations. First, the samples in the GSE31210 dataset were relatively small in size, suggesting the need for additional large external datasets to validate the risk scores. Second, experimental studies involving five predicted genes are needed to investigate the comprehensive molecular mechanisms of LUAD initiation and development.

## Conclusion

To summarize, the current work developed and validated for the first time a unique risk score prediction model based on five genes related to fatty acid metabolism. Further, we analyzed the differences in clinical characteristics, chemotherapy and targeted treatment sensitivity, and infiltration of immune cells among individuals with LUAD at higher and lower risk, which might facilitate the treatment of patients. In brief, not only does this risk score model enable the prognosis of patients with LUAD, but also provides new insights into the carcinogenesis and therapeutic strategies of LUAD.

## Supplementary Information

**Additional file 1.** **Table S1**: Clinical information of patients with LUAD from TCGA cohort (n = 513). **Table S2**: Clinical information of patients with LUAD from GSE31210 cohort (n = 226). **Table S3**: The gene symbol of 309 fatty acid-related genes. **Table S4**: Details of five genes for constructing the prognostic risk score model. **Figure S1**: The expression levels of five model genes. (A, F) LDHA; (B, G) ALDOA; (C, H) CYP4B1; (D, I) DPEP2; (E, J) HPGDS.

## Data Availability

The datasets used in this investigation are available through the TCGA database (https://tcga-data.nci.nih.gov/) and the GEO database (http://www.ncbi.nlm.nih.gov /geo/).

## Abbreviations

ACSL4: Acyl-coA synthetase long chain family member 4
ACOT11: Acyl-coA thioesterase 11
ASBG1: Acyl-CoA synthetase bubblegum family member 1
ALDH2: Aldehyde dehydrogenase 2
ALDOA: Aldolase A
AUC: Area under the receiver operating characteristic curve
CA4: Carbonic anhydrase 4
CIBERSORT: Cell-type identification by estimating relative subsets of RNA transcript
CYP4B1: Cytochrome P450 family 4 subfamily B member 1
DEGs: Differentially expressed genes
DPEP2: Dipeptidase 2
ELOVL6: ELOVL fatty acid elongase 6
FDR: False discovery rate
FC: Fold change
GEO: Gene expression omnibus
GO: Gene ontology
GSVA: Gene set variation analysis
IC50: Half-maximal inhibitory concentration
HR: Hazard ratio
HPGDS: Hematopoietic prostaglandin D synthase
HLA: Human leukocyte antigen
KEGG: Kyoto Encyclopedia of Genes and Genomes
LASSO: Least absolute shrinkage and selection operator
LUAD: Lung adenocarcinoma
MIF: Macrophage migration inhibitory factor
MSigDB: Molecular signatures database
OS: Overall survival
PCA: Principal component analysis
PFS: Progression free survival
PTGDS: Prostaglandin D2 synthase
ROC: Receiver operating characteristic
ssGSEA: Single-sample gene set enrichment analysis
TCGA: The cancer genome atlas
TIDE: Tumor immune dysfunction and exclusion
TME: Tumor microenvironment

## References

[1] Siegel RL, Miller KD, Fuchs HE (2021). Cancer statistics, 2021. CA Cancer J Clin.

[2] Barta JA, Powell CA, Wisnivesky JP (2019). Global epidemiology of lung cancer. Ann Glob Health.

[3] Bi KW, Wei XG, Qin XX (2020). BTK has potential to be a prognostic factor for lung adenocarcinoma and an indicator for tumor microenvironment remodeling: a study based on TCGA data mining. Front Oncol.

[4] Lin JJ, Cardarella S, Lydon CA (2016). Five-year survival in EGFR-mutant metastatic lung adenocarcinoma Treated with EGFR-TKIs. J Thorac Oncol.

[5] Bose S, Le A (2018). Glucose metabolism in cancer. Adv Exp Med Biol.

[6] Peng H, Wang Y, Luo W (2020). Multifaceted role of branched-chain amino acid metabolism in cancer. Oncogene.

[7] Liang C, Wang X, Zhang Z (2020). ACOT11 promotes cell proliferation, migration and invasion in lung adenocarcinoma. Transl Lung Cancer Res.

[8] Zhang Y, Li S, Li F (2021). High-fat diet impairs ferroptosis and promotes cancer invasiveness via downregulating tumor suppressor ACSL4 in lung adenocarcinoma. Biol Direct.

[9] He D, Cai L, Huang W (2021). Prognostic value of fatty acid metabolism-related genes in patients with hepatocellular carcinoma. Aging.

[10] Hänzelmann S, Castelo R, Guinney J (2013). GSVA: gene set variation analysis for microarray and RNA-seq data. BMC Bioinform.

[11] Geeleher P, Cox N, Huang RS (2014). pRRophetic: an R package for prediction of clinical chemotherapeutic response from tumor gene expression levels. PLoS ONE.

[12] Newman AM, Liu CL, Green MR (2015). Robust enumeration of cell subsets from tissue expression profiles. Nat Methods.

[13] Gentles AJ, Newman AM, Liu CL (2015). The prognostic landscape of genes and infiltrating immune cells across human cancers. Nat Med.

[14] Ru B, Wong C, Tong Y (2019). TISIDB: an integrated repository portal for tumor-immune system interactions. Bioinformatics.

[15] Charoentong P, Finotello F, Angelova M (2017). Pan-cancer immunogenomic analyses reveal genotype-immunophenotype relationships and predictors of response to checkpoint blockade. Cell Rep.

[16] Ding C, Shan Z, Li M (2021). Characterization of the fatty acid metabolism in colorectal cancer to guide clinical therapy. Mol Ther Oncolytics.

[17] Jiang P, Gu S, Pan D (2018). Signatures of T cell dysfunction and exclusion predict cancer immunotherapy response. Nat Med.

[18] Faubert B, Solmonson A, Deberardinis RJ (2020). Metabolic reprogramming and cancer progression. Science.

[19] Zhao Y, Zhang J, Wang S (2021). Identification and validation of a nine-gene amino acid metabolism-related risk signature in HCC. Front Cell Dev Biol.

[20] Zhang L, Zhang Z, Yu Z (2019). Identification of a novel glycolysis-related gene signature for predicting metastasis and survival in patients with lung adenocarcinoma. J Transl Med.

[21] Yang Y, Su D, Zhao L (2014). Different effects of LDH-A inhibition by oxamate in non-small cell lung cancer cells. Oncotarget.

[22] Wang X, Xie X, Zhang Y (2022). Hippocalcin-like 1 is a key regulator of LDHA activation that promotes the growth of non-small cell lung carcinoma. Cell Oncol (Dordr).

[23] Li X, Lu P, Li B (2016). Sensitization of hepatocellular carcinoma cells to irradiation by miR-34a through targeting lactate dehydrogenase-A. Mol Med Rep.

[24] Li L, Kang L, Zhao W (2017). miR-30a-5p suppresses breast tumor growth and metastasis through inhibition of LDHA-mediated Warburg effect. Cancer Lett.

[25] Liu X, Yang Z, Chen Z (2015). Effects of the suppression of lactate dehydrogenase A on the growth and invasion of human gastric cancer cells. Oncol Rep.

[26] Lei W, Kang W, Nan Y (2018). The downregulation of miR-200c promotes lactate dehydrogenase A expression and non-small cell lung cancer progression. Oncol Res.

[27] De Oliveira PSN, Coutinho LL, Cesar ASM (2019). Co-expression networks reveal potential regulatory roles of miRNAs in fatty acid composition of nelore cattle. Front Genet.

[28] Dai L, Pan G, Liu X (2018). High expression of ALDOA and DDX5 are associated with poor prognosis in human colorectal cancer. Cancer Manag Res.

[29] Ji S, Zhang B, Liu J (2016). ALDOA functions as an oncogene in the highly metastatic pancreatic cancer. Cancer Lett.

[30] Saito Y, Takasawa A, Takasawa K (2020). Aldolase A promotes epithelial-mesenchymal transition to increase malignant potentials of cervical adenocarcinoma. Cancer Sci.

[31] Liu X, Jia Y, Shi C (2021). CYP4B1 is a prognostic biomarker and potential therapeutic target in lung adenocarcinoma. PLoS ONE.

[32] Rittchen S, Heinemann A (2019). Therapeutic potential of hematopoietic prostaglandin D synthase in allergic inflammation. Cells.

[33] Zhang J, Zhang Y, Gong H (2017). Genetic mapping using 1.4M SNP array refined loci for fatty acid composition traits in Chinese Erhualian and Bamaxiang pigs. J Anim Breed Genet.

[34] Wang J, Mak O (2011). Induction of apoptosis in non-small cell lung carcinoma A549 cells by PGD_2_ metabolite, 15d-PGJ_2_. Cell Biol Int.

[35] Panossian A, Seo EJ, Efferth T (2019). Effects of anti-inflammatory and adaptogenic herbal extracts on gene expression of eicosanoids signaling pathways in isolated brain cells. Phytomedicine.

[36] Miyata J, Fukunaga K, Kawashima Y (2019). Dysregulated fatty acid metabolism in nasal polyp-derived eosinophils from patients with chronic rhinosinusitis. Allergy.

[37] Lee JM, Lee MH, Garon E (2017). Phase I trial of intratumoral injection of gene-modified dendritic cells in lung cancer elicits tumor-specific immune responses and CD8 T-cell infiltration. Clin Cancer Res.

[38] Hegde PS, Karanikas V, Evers S (2016). The where, the when, and the how of immune monitoring for cancer immunotherapies in the era of checkpoint inhibition. Clin Cancer Res.

[39] Salamon P, Mekori Y, Shefler I (2020). Lung cancer-derived extracellular vesicles: a possible mediator of mast cell activation in the tumor microenvironment. Cancer Immunol Immunother.

[40] Hao L, Zhang J, Di Y (2018). Prognostic value of white blood cells detected for the first time after adjuvant chemotherapy in primary operable non-small cell lung cancer. Technol Cancer Res Treat.

[41] Shau HY, Kim A (1988). Suppression of lymphokine-activated killer induction by neutrophils. J Immunol.

