# Supplementary material for: Characterization of the fatty acid metabolism-related genes in lung adenocarcinoma to guide clinical therapy
Source: BMC Pulm Med. 2022 Dec 23;22:486. doi: 10.1186/s12890-022-02286-3 (PMC9784226; doi:10.1186/s12890-022-02286-3)

**Supplementary Table 1**. Clinical information of patients with LUAD from TCGA cohort (n=513)

| **Clinical characteristic** | **N** | **%** |
| --- | --- | --- |
| **Age** |  |  |
| ≤65 | 238 | 46.4 |
| >65 | 256 | 49.9 |
| NA | 19 | 3.7 |
| **Gender** |  |  |
| Female | 276 | 53.8 |
| Male | 237 | 46.2 |
| **TNM Stage** |  |  |
| I | 274 | 53.4 |
| II | 121 | 23.6 |
| III | 84 | 16.4 |
| IV | 26 | 5.1 |
| NA | 8 | 1.5 |
| **T stage** |  |  |
| T1 | 168 | 32.7 |
| T2 | 276 | 53.8 |
| T3 | 47 | 9.2 |
| T4 | 19 | 3.7 |
| NA | 3 | 0.6 |
| **N stage** |  |  |
| N0 | 330 | 64.3 |
| N1 | 95 | 18.5 |
| N2 | 74 | 14.4 |
| N3 | 2 | 0.4 |
| NA | 12 | 2.4 |
| **M stage** |  |  |
| M0 | 344 | 67.0 |
| M1 | 25 | 4.9 |
| NA | 144 | 28.1 |
| **Survival time** |  |  |
| Reported | 504 | 98.2 |
| NA | 9 | 1.8 |
| **Survival status** |  |  |
| Alive | 326 | 63.5 |
| Dead | 187 | 36.5 |

**Supplementary Table 2.** Clinical information of patients with LUAD from GSE31210 cohort (n=226)

| **Clinical characteristic** | **N** | **%** |
| --- | --- | --- |
| **Age** |  |  |
| ≤65 | 176 | 77.9 |
| >65 | 50 | 22.1 |
| **Gender** |  |  |
| Female | 121 | 53.5 |
| Male | 105 | 46.5 |
| **TNM Stage** |  |  |
| I | 168 | 74.3 |
| II | 58 | 25.7 |
| **Survival time** |  |  |
| Reported | 226 | 100.0 |
| **Survival status** |  |  |
| Alive | 191 | 84.5 |
| Dead | 35 | 15.5 |

**Supplementary Table 3.** The gene symbol of 309 fatty acid-related genes

| FADS2 | ACACA | CYP2C8 | CYP4F3 | AUH | PRXL2B |
| --- | --- | --- | --- | --- | --- |
| RAP1GDS1 | ALDH1A1 | ECI1 | MAPKAPK2 | D2HGDH | DECR2 |
| SDHA | ACAA1 | CYP2J2 | CBR4 | SLC22A5 | SLC25A20 |
| ALDH2 | CA4 | RXRA | MCAT | H2AZ1 | ACOX1 |
| ACSBG1 | OLAH | THEM4 | ODC1 | SMS | ACSL6 |
| HSD17B4 | PRKAA2 | FABP1 | ALAD | XIST | ACSL4 |
| HACD1 | ACOT7 | PDHB | CIDEA | ALOX12B | AQP7 |
| HSD17B3 | ACOT8 | PTGR2 | ELOVL4 | ELOVL5 | IL4I1 |
| PTGES2 | ABCD1 | ACOXL | CA2 | NUDT7 | ACADL |
| DLD | ACSL3 | NDUFAB1 | RDH11 | ALDOA | PTGIS |
| CBR1 | GLUL | CBR3 | AOC3 | VNN1 | ALDH3A2 |
| ADIPOR2 | ACOT9 | MCEE | GGT1 | CYP4B1 | REEP6 |
| HPGD | NCAPH2 | PTGES3 | UROS | MMUT | HMGCL |
| CD1D | ADH5 | CRYZ | CYP2C19 | ACO2 | ALDH7A1 |
| ERP29 | HACD2 | PCTP | LDHA | UGDH | ACAD11 |
| PTGES | OSTC | THEM5 | ACADM | HSD17B12 | INMT |
| AADAT | CYP1B1 | CPT1B | ACOT13 | MDH2 | ACACB |
| PDHA1 | CYP4F22 | LTA4H | HADHB | PPT1 | CPT1C |
| ACAA2 | FADS1 | CYP8B1 | ADH6 | DPEP2 | CYP4F11 |
| ACSF3 | HMGCS2 | ADH1C | ACADSB | EPHX2 | ADH1B |
| MAOA | IDH3G | HSD17B8 | ACSL1 | ACOT6 | ACOX2 |
| PRDX6 | ALOX15 | BLVRA | ACAT1 | PON2 | DECR1 |
| CD36 | MECR | SUCLG2 | G0S2 | PSME1 | ENO2 |
| PRKAG2 | CPOX | THRSP | ELOVL1 | ACOT4 | CYP4F2 |
| ACADVL | HSD17B11 | ACOX3 | CROT | PTGS2 | IDI1 |
| ELOVL2 | ACSM6 | ECH1 | GABARAPL1 | PTPRG | ALDH9A1 |
| ALOX15B | HACD4 | CYP1A2 | ALOX5AP | HAO2 | HIBCH |
| PON1 | FAAH | GCDH | NSDHL | ACOT2 | LTC4S |
| MDH1 | CYP2U1 | ELOVL3 | FMO1 | SLC27A3 | ELOVL7 |
| PECR | NTHL1 | PON3 | ACAT2 | SERINC1 | ALOX12 |
| PPT2 | MIX23 | TDO2 | ALOX5 | DLST | MMAA |
| GPX1 | SDHD | ACADS | PTS | HSD17B10 | NBN |
| ENO3 | HSPH1 | SCD5 | TECRL | ACLY | LGALS1 |
| ACBD4 | KMT5A | YWHAH | GPX4 | TECR | GAPDHS |
| CA6 | MID1IP1 | GGT5 | CPT1A | HMGCS1 | MGLL |
| CEL | ALDH1B1 | TBXAS1 | FAAH2 | ECI2 | SLC27A2 |
| CYP4F8 | PPARD | AMACR | ECHS1 | BPHL | PPARA |
| ACBD6 | HTD2 | PCCA | GSTZ1 | METAP1 | HSP90AA1 |
| MORC2 | SUCLA2 | ACOT11 | ACSS1 | APEX1 | MLYCD |
| SDHC | ADH4 | PTGS1 | HACD3 | ELOVL6 | CYP1A1 |
| UBE2L6 | UROD | EPHX1 | IDH3B | MIF | PCBD1 |
| CYP2C9 | PTGDS | ACSBG2 | GRHPR | RETSAT | SLC25A17 |
| CYP4A22 | HADHA | ACSF2 | FH | NUDT19 | BCKDHB |
| ADH1A | ABCC1 | ALDH3A1 | ETFDH | HPGDS | DBI |
| DPEP3 | PTGR1 | ME1 | ACSM3 | HSDL2 | SUCLG1 |
| ACBD5 | DHCR24 | FASN | PLA2G4A | ADSL | CPT2 |
| AWAT1 | HCCS | DPEP1 | ACAD10 | PCCB | ACOT12 |
| ALOXE3 | FABP2 | GPX2 | HADH | ACSL5 | HACL1 |
| SLC25A1 | GPD1 | GAD2 | ACBD7 | SCD | ADH7 |
| EHHADH | PHYH | AKR1C3 | SCP2 | RDH16 | GPD2 |
| TP53INP2 | IDH1 | S100A10 | CRAT | HSD17B7 | CYP4A11 |
| ACOT1 | PRKAB2 | BMPR1B |  |  |  |

**Supplementary Table 4.** Details of five genes for constructing the prognostic risk score model

| **Gene** | **Ensemble ID** | **Location** | **HR (95%CI)** | **Coefficient** |
| --- | --- | --- | --- | --- |
| LDHA | ENSG00000134333 | chr11:18,394,560-18,408,425 | 1.9287 | 0.3635 |
| ALDOA | ENSG00000149925 | chr16:30,064,164-30,070,457 | 1.5089 | 0.0222 |
| CYP4B1 | ENSG00000142973 | chr1:46,757,838-46,819,413 | 0.8603 | -0.0483 |
| DPEP2 | ENSG00000167261 | chr16:67,987,390-68,000,644 | 0.6661 | -0.0026 |
| HPGDS | ENSG00000163106 | chr4:94,298,535-94,342,876 | 0.7684 | -0.0653 |

**Supplementary Figure 1 The expression levels of five model genes.**

(A, F) LDHA; (B, G) ALDOA; (C, H) CYP4B1; (D, I) DPEP2; (E, J) HPGDS.


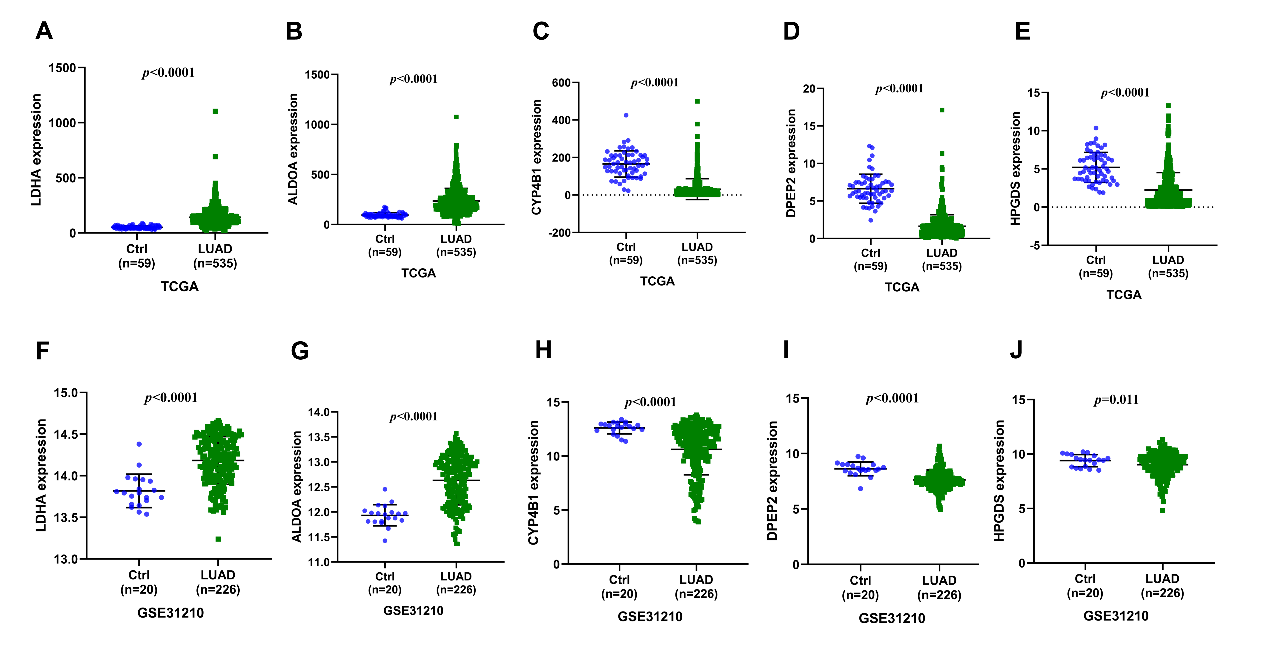

Supplement: Supplementary file 1 — Additional file 1. Table S1: Clinical information of patients with LUAD from TCGA cohort (n = 513). Table S2: Clinical information of patients with LUAD from GSE31210 cohort (n = 226). Table S3: The gene symbol of 309 fatty acid-related genes. Table S4: Details of five genes for constructing the prognostic risk score model. Figure S1: The expression levels of five model genes. (A, F) LDHA; (B, G) ALDOA; (C, H) CYP4B1; (D, I) DPEP2; (E, J) HPGDS. [file 12890_2022_2286_MOESM1_ESM.docx]
